# Fluoroscopy: Taking a closer look at joint motion in osteoarthritis

**DOI:** 10.1016/j.ostima.2024.100240

**Published:** 2024-06-22

**Authors:** N.B.J. Dur, M.G.H. Wesseling, E.M. Macri, J. Runhaar

**Affiliations:** a Department of Orthopaedics and Sports Medicine, Erasmus MC University Medical Center Rotterdam, PO-Box 2040, 3000CA Rotterdam, the Netherlands; b Department of Radiology & Nuclear Medicine, Erasmus MC University Medical Center Rotterdam, PO-Box 2040, 3000CA Rotterdam, the Netherlands; c Department of Biomechanical Engineering, Delft University of Technology, Mekelweg 2, 2628CD Delft, the Netherlands; d Department of General Practice, Erasmus MC University Medical Center Rotterdam, PO-box 2040, 3000CA Rotterdam, the Netherlands

**Keywords:** Fluoroscopy, Biomechanics, Kinematics, Osteoarthritis

## Abstract

**Background:**

Knowledge of joint biomechanics is crucial for understanding the pathophysiology of osteoarthritis (OA). Biomechanical changes, especially in early-stage OA, are subtle and therefore require highly accurate and precise ways of measuring joint loading (i.e., forces) and kinematics (i.e., motion). Most OA research investigating kinematics to date using conventional measurement methods, has been restricted either to measures of functional, weight-bearing activities with limited precision, or to highly precise measures of non-functional activities. X-ray fluoroscopy, however, is a medical imaging modality that can be used to precisely measure high-speed dynamic human movement in vivo under weight-bearing conditions.

**Objective:**

To illustrate the potential of fluoroscopy to measure human movement and study joint biomechanics in OA by highlighting several exemplary applications in studies on knee OA.

**Design:**

We searched PubMed to identify a selection of relevant studies with fluoroscopic evaluation of joint mechanics in individuals with, or at risk of developing, knee OA.

**Results:**

Fluoroscopy has been utilized in biomechanical studies in populations with early stages of knee OA or at risk of developing knee OA, as well as in studies evaluating conservative treatment strategies for knee OA. Due to its ability to capture highly accurate and precise kinematic measures, fluoroscopy holds great promise in providing new insights in the role of biomechanics in OA pathogenesis.

**Conclusions:**

The use of fluoroscopy in larger cohorts and longitudinal (interventional) studies may lead to more robust and generalizable results and could clarify the mediation pathways of joint biomechanics in the onset and progression of OA.

## Highlights

• Fluoroscopy allows for non-invasive, high-speed, in vivo measurement of joint motion during weight-bearing conditions with greater accuracy and precision compared to more conventional methods such as optical motion capture.
• Fluoroscopy is particularly superior in assessment of kinematics during explosive movements, in individuals with excess adipose tissue, and for rotations in the non-sagittal plane.
• Since small kinematic changes are thought to play an important role in OA pathogenesis, fluoroscopy’s superior accuracy and precision are especially relevant in biomechanical research in (early-stage) OA.

## Introduction

Joint biomechanics is considered one of the key etiological factors in the onset and progression of osteoarthritis (OA). Hence, knowledge of biomechanics is crucial for understanding OA pathophysiology and for developing treatment strategies to slow down or even prevent OA onset and progression and to alleviate symptoms. According to a framework for in vivo OA pathomechanics developed by Andriacchi et al. (2004), kinematic changes in early-stage OA cause loads (i.e., forces; Table 1) to be shifted towards less frequently loaded areas of the cartilage that cannot accommodate these loads, initiating a cascade of degenerative changes which eventually result in irreversible structural changes within the joint [1]. A substantial amount of research has been conducted in patients with various stages of OA in an attempt to characterize and distinguish healthy from pathological movement patterns. In particular, a special interest in identifying these patterns in early stages of OA has emerged, as this could offer a window of opportunity in which the changes may still be reversible, and preventative or disease-modifying interventions may be more effective [2].

Accurately evaluating biomechanics to clarify their role in OA is methodologically challenging. One major challenge is that these biomechanical changes, especially in early-stage OA, are subtle and therefore require highly accurate and precise ways of measuring joint loading. Currently, there is no direct method to non-invasively measure in vivo joint loading. It is possible to use biomechanical computer models to estimate joint loading, but doing so requires accurate information about joint kinematics (i.e., motion; Table 1) measured during functional, weight-bearing activities. Hence, dynamic joint kinematics under weight-bearing conditions have been studied to estimate in vivo joint loading using various methods. Use of intra-cortical pins, directly inserted into the bone, provides accurate kinematic measurements, but may affect subjects’ natural gait pattern due to pain and impingement [3], and does not get around the invasive nature of the measurement. To measure kinematics non-invasively under weight-bearing conditions, optical motion capture (OMC) is commonly used, in which retroreflective skin-mounted markers are tracked in 3D space to evaluate joint motion. However, movement of skin-mounted markers relative to the underlying bone, known as soft tissue artifacts, causes substantial errors. These errors have been reported to be up to 9° in knee rotations during walking and even up to 24° during an open-chain knee flexion [4]. Static and dynamic magnetic resonance (MR) or computed tomography (CT) imaging avoids errors due to soft tissue artifacts, by directly imaging bone orientations. These imaging modalities, however, have only a limited ability to measure functional movements, since the prone or supine positioning required for most systems complicates measurement under weight-bearing conditions, and confined imaging environments limit possible range of motion. Most OA research investigating biomechanics to date has therefore been restricted to either measures of functional, weight-bearing activities with limited precision, or highly precise measures of non-functional activities. By uniting these previously mutually exclusive properties, fluoroscopy has been recognized as a promising solution to this problem and is attracting increased interest in studies on human movement.

The aim of this narrative mini-review is to illustrate the potential of using fluoroscopy to measure human movement and study joint biomechanics in OA and to highlight several exemplary applications in studies on OA of the knee. Furthermore, we will discuss some future possibilities of this technique and its potential in OA research.

A literature search was conducted in PubMed to identify a selection of relevant studies with fluoroscopic evaluation of joint mechanics in individuals with or at risk of developing knee OA. Articles were screened and selected by one author based on relevance and the final selection was discussed and confirmed by all authors.

### Fluoroscopic measurement of joint kinematics

**Fig. 1:**
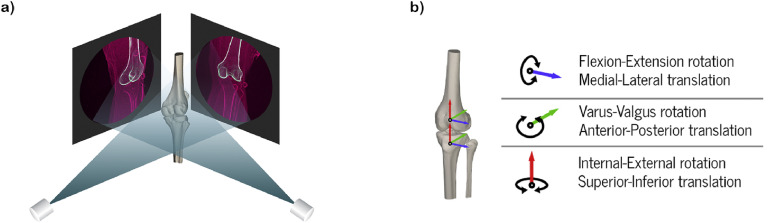
Deriving kinematic measurements from fluoroscopy images.

X-ray fluoroscopy is a medical imaging modality that can be used to non-invasively measure high-speed, in vivo, dynamic human movement, with the ability to do so under weight-bearing conditions [5]. Technical advances in recent years have improved the technique's measuring capabilities and expanded its applicability in human movement research. In particular, fluoroscopy instruments are now capable of capturing a larger field of view (panel sizes up to 50 × 50 cm [6]) at higher frame rates (typically up to 100 frames per second [7,8]), using a radiation dose equivalent to less than a month of background radiation [7]. Although fluoroscopy yields two-dimensional (2D) images, 3D bone orientation can be determined by registering a separately acquired 3D geometric model to the 2D fluoroscopy images (Fig. 1a). A number of studies have used fluoroscopy to measure biomechanics in joint arthroplasties, in which the known 3D shape of the metal arthroplasty components is registered to the 2D fluoroscopy images. To utilize this technique in native joints, a personalized 3D bone model obtained from CT or MR images is required. Due to innovations in image processing algorithms, obtaining these models has become more automated, faster, and more accurate, making them more feasible for use in biomechanical studies in native joints. By assigning local coordinate systems based on the anatomic axes of the 3D bone models (Fig. 1b), joint movement can be quantified in six degrees of freedom (i.e., three translations and three rotations; Table 1) with far greater accuracy and precision than conventional methods, such as OMC. Using a single (monoplanar) fluoroscopy setup to evaluate knee kinematics, average root-mean-squared errors of 0.53 mm for in-plane translations (Table 1), 1.59 mm for out-of-plane translations and 0.54° for rotations have been reported [9]. Adding a second fluoroscope yields a biplanar setup, in which the two instruments are positioned at an angle to each other, further increasing accuracy, especially for out-of-plane translations (root-mean-squared error of 0.69 mm) [10]. A wide variety of functional movements can be evaluated using fluoroscopy, including squats [6,[11], [12], [13], [14]], step-ups [12], kneeling [12], and pivoting [6]. Combining the fluoroscopy setup with a treadmill enables the joint of interest to remain within the system's field of view throughout all phases of gait and thus allows for assessment of level walking or running [7,8,10,15]. By tilting the treadmill, higher-demanding tasks such as decline treadmill walking can be assessed [7,16]. Especially in more explosive movements, in which error due to soft tissue artifacts is more substantial, fluoroscopy enables more reliable kinematic measurements than OMC-based methods.

**a)** Registration of a personalized 3D bone model with biplanar 2D fluoroscopy images. A projection of the femur bone model (white) is matched with the femur on fluoroscopy. **b)** Assignment of local coordinate systems based on the anatomical axes of the 3D bone models. This way joint movements can be quantified in six degrees-of-freedom (3 translations and 3 rotations).

**Table 1:** Overview of biomechanical terms and definitions.

Term:	Definition:
Joint loading	Mechanics concerning the force acting between articulating bones.
Joint kinematics	Mechanics concerning the motion between articulating bones.
Six degrees-of-freedom	Six mechanical degrees of movement, consisting of three perpendicular directions of translation (antero-posterior, medial-lateral, proximal-distal) and rotations about three perpendicular axes (frontal, sagittal, axial)
In-plane and out-of-plane kinematics	Motions in the plane orthogonal and parallel to the direction of the fluoroscopy beam, respectively.
Contact point	Location of the center of the cartilage-to-cartilage contact between articulating bones.

### Examples of fluoroscopy in knee OA research

With its superior accuracy and precision, fluoroscopy has unlocked the potential to study joint movement patterns and quantify weight-bearing kinematics in early stages of knee OA pathogenesis.

Obesity is one of the biggest risk factors for developing knee OA [17]. To date, however, biomechanics studies using OMC-based measurements have been constrained by considerable soft tissue artifacts due to the considerable amount of adipose tissue, which limits measurement reliability and impedes longitudinal studies after weight loss programs. More reliable measurements of joint kinematics obtained from fluoroscopy were used to analyze cartilage-to-cartilage contact locations in the knee during gait in obese individuals without radiographic signs of knee OA and showed a pattern of increased medial-lateral contact point (Table 1) excursions compared to non-obese individuals [15]. Such findings may help to explain the high prevalence of knee OA in this population, as well as to explain where OA occurs in the knee.

Other populations at risk of early development and rapid progression of knee OA that have been studied using fluoroscopy include individuals with an anterior cruciate ligament (ACL) rupture [13,14] or with a medial meniscal root tear [7]. The stabilizing role of these structures in anteroposterior translation and internal tibial rotation is widely accepted, and the kinematics of ACL deficiency, with or without concomitant meniscal injury, are well known in terms of injury pattern and manual tests (e.g., pivot shift test) [18]. How injury to these stabilizing structures contributes to onset and progression of knee OA, however, is still not completely understood. Studies using fluoroscopy to study biomechanics in ACL-deficient knees have found differences in kinematic patterns compared to healthy controls [13,14]. Although discrepancies exist in the reported anatomical plane in which these differences occur, they are consistently reported to deviate the most in the medial compartment. This might explain the high incidence of OA in the medial compartment seen in chronic ACL-deficiency [19].

In studies conducted in individuals with established early-stage knee OA, defined as Kellgren-Lawrence grade 1 or 2, differences in tibial rotation and lateral contact point location compared to healthy knees were found [6,11]. These patterns are similar to kinematic patterns observed in knees with later stages of OA [11], which may suggest that there are specific kinematic changes that persist across all stages of knee OA. Rotations in the non-sagittal planes are particularly unreliable when measured using OMC, since errors caused by soft tissue artifacts are relatively large, compared to the limited range of motion seen in these planes in most functional tasks [4]. Therefore, these patterns may not have been detectable using OMC.

Besides more accurate measurements of six degrees of freedom kinematics, more accurate joint contact point locations can be derived through fluoroscopy as another kinematic measure, and their trajectory can be characterized throughout the full range of motion. Increased excursions and/or velocities of contact points during motion may suggest joint instability, and observed contact point trajectories may explain specific patterns of cartilage degradation observed in knee OA. By determining the distance between 3D femoral and tibial bones, proximity-based algorithms have been used to estimate tibiofemoral contact points for both the medial and lateral compartments [12]. In addition to bone geometry, cartilage geometry can be taken into account by incorporating MR-based, subject-specific cartilage surface models, which are registered to the bone models to derive cartilage contact points or estimate cartilage stress profiles [15,16]. This may yield more accurate contact point locations, especially in lower flexion angles, by taking variations in cartilage thickness within or between participants into account [20].

Using fluoroscopy, similar proximity-based measurements have been used to evaluate the efficacy of conservative treatments such as medial compartment unloader braces in patients with advanced medial knee OA during in vivo, weight-bearing conditions [8]. The small but significant increase in dynamic joint space in the medial compartment and reduction of pain when wearing an unloader brace reported in this study, supports the underlying premise that unloader braces can alleviate symptoms by unloading the affected compartment of the knee. By enabling assessment of changes in tibiofemoral joint space during weight-bearing activities, fluoroscopy offers a more objective measurement to assess the underlying mechanisms by which unloader braces affect patient pain and functional outcome, which can provide a base for improving brace design and patient care.

### Remaining challenges and future directions

While fluoroscopy surpasses more conventional methods of measuring dynamic, in vivo, weight-bearing joint kinematics in terms of accuracy and precision, it also has some limitations. Since participants are exposed to a small amount of radiation, fluoroscopy systems must be used in isolated radiation-compliant facilities by trained personnel. Regarding fluoroscopy's applicability, the type of dynamic tasks and number of joints that can be captured simultaneously is constrained by the fluoroscope's limited field of view compared to OMC, in which full-body kinematics can be captured in a much larger volume. These two technologies can be combined to benefit from their complementary strengths. Further improvements in image detectors in terms of field of view, spatial resolution and dynamic range will allow for greater anatomical coverage and lower amounts of radiation needed for adequate image contrast. Moreover, systems with higher frame rates, already reported to be up to 250 frames per second [10], will enable assessment of even faster movements.

As for the feasibility of fluoroscopy, the substantial costs and the time necessary for processing the images make it less feasible for larger cohort studies, which is reflected by the mostly small sample sizes and cross-sectional study designs published to date. There are, however, major developments in progress in computer processing speed and automating of image processing algorithms, which should lead to faster and less labor-intensive processing. Eventually, this improved feasibility will enable researchers to deploy fluoroscopy's superior accuracy and precision in measuring functional, weight-bearing activities in larger cohorts and longitudinal (interventional) studies, which could lead to more robust and more generalizable results and could clarify the mediation pathways of joint biomechanics in the onset and progression of OA.

## Funding

This research did not receive any specific grant form funding agencies in the public, commercial, or non-for-profit sectors.

## CRediT authorship contribution statement

**N.B.J. Dur:** Conceptualization, Methodology, Investigation, Writing – original draft, Writing – review & editing, Visualization, Project administration. **M.G.H. Wesseling:** Writing – review & editing, Supervision. **E.M. Macri:** Writing – review & editing, Supervision. **J. Runhaar:** Writing – review & editing, Supervision.

## Declaration of competing interest

JR delivered a keynote speech on the topic of this manuscript at the IWOAI 2023 and was compensated for registration and housing. The remaining authors declare no conflicts of interest that are relevant to the content of this article.
